# Information Transfer in Linear Multivariate Processes Assessed through Penalized Regression Techniques: Validation and Application to Physiological Networks

**DOI:** 10.3390/e22070732

**Published:** 2020-07-01

**Authors:** Yuri Antonacci, Laura Astolfi, Giandomenico Nollo, Luca Faes

**Affiliations:** 1 Department of Computer, Control and Management Engineering, Sapienza University of Rome, 00185 Rome, Italy; laura.astolfi@uniroma1.it; 2 Istituto di Ricovero e Cura a Carattere Scientifico (IRCCS) Fondazione Santa Lucia, 00179 Rome, Italy; 3 Department of Industrial Engineering, University of Trento, 38123 Trento, Italy; giandomenico.nollo@unitn.it; 4 Department of Engineering, University of Palermo, 90128 Palermo, Italy; luca.faes@unipa.it

**Keywords:** information dynamics, partial information decomposition, entropy, conditional transfer entropy, network physiology, multivariate time series analysis, State–space models, vector autoregressive model, penalized regression techniques, linear prediction

## Abstract

The framework of information dynamics allows the dissection of the information processed in a network of multiple interacting dynamical systems into meaningful elements of computation that quantify the information generated in a target system, stored in it, transferred to it from one or more source systems, and modified in a synergistic or redundant way. The concepts of information transfer and modification have been recently formulated in the context of linear parametric modeling of vector stochastic processes, linking them to the notion of Granger causality and providing efficient tools for their computation based on the state–space (SS) representation of vector autoregressive (VAR) models. Despite their high computational reliability these tools still suffer from estimation problems which emerge, in the case of low ratio between data points available and the number of time series, when VAR identification is performed via the standard ordinary least squares (OLS). In this work we propose to replace the OLS with penalized regression performed through the Least Absolute Shrinkage and Selection Operator (LASSO), prior to computation of the measures of information transfer and information modification. First, simulating networks of several coupled Gaussian systems with complex interactions, we show that the LASSO regression allows, also in conditions of data paucity, to accurately reconstruct both the underlying network topology and the expected patterns of information transfer. Then we apply the proposed VAR-SS-LASSO approach to a challenging application context, i.e., the study of the physiological network of brain and peripheral interactions probed in humans under different conditions of rest and mental stress. Our results, which document the possibility to extract physiologically plausible patterns of interaction between the cardiovascular, respiratory and brain wave amplitudes, open the way to the use of our new analysis tools to explore the emerging field of Network Physiology in several practical applications.

## 1. Introduction

Physiological systems such as the cerebral, cardiac, vascular and respiratory system exhibit a dynamic activity which results from the continuous modulation of multiple control mechanisms and changes transiently across different physiological states. Accordingly, the human body can be modeled as an ensemble of complex physiological systems, each with its own regulatory mechanisms, that dynamically interact to preserve the physiological functions [1]. These interactions are commonly studied in a non-invasive way by recording physiological signals that are subsequently elaborated to extract time series of interest which reflect the dynamic state of the system under analysis [2,3]. Many studies in the literature have provided strong evidence about the existence of a relationship between the properties of time series extracted and the physiological functions, even if most of these evidences come from the analysis of the dynamics within a single system (i.e., variability of heart rate, activity or connectivity within brain networks [4,5]) or at most between two systems (cardiovascular, cardio-respiratory and brain–heart interactions [6,7]). Only recently, with the introduction of the concept of network physiology grounded on a system-wide integration approach, it has been possible to analyze the physiological interactions in a fully multivariate fashion. With this approach, the various physiological systems that compose the human organism are considered to be the nodes of a complex network [8]. Nevertheless, identifying a network comprised of different dynamic physiological systems is a non-trivial task that requires the development of methodological approaches able to take into account the intrinsically multivariate nature of the network, and to describe the different aspects of network activity and connectivity dealing with complex dynamics and intricate topological structures.

Recent studies in the context of information theory have shown how the information processing in a network of multiple interacting dynamical systems, described by multivariate stochastic processes, can be dissected into basic elements of computation defined with the so-called framework of information dynamics [9]. These elements essentially reflect the new information produced at each moment in time about a target system in the network, the information stored in the target system, the information transferred to it from other connected systems and the modification of the information flowing from multiple sources to the target [10]. In particular, the information transfer defines the information that a group of systems designed as “sources” provide about the present state of the target [11]; information modification is strongly related to the concept of redundancy and synergy between two source systems sharing information about a target system, which refers to the existence of common information about the target that can be recovered when the sources are used separately (redundancy) or when they are used jointly (synergy) [12]. Thus, positive values of information modification indicate net synergy, which reflects the concept of information independence of the sources. On the other hand, negative values of information modification indicate redundancy, which reflects the fact that no additional information is conveyed about the target system when the two sources are considered together rather than in isolation [13]. Operational definitions of these concepts have been recently proposed, also showing how—for Gaussian processes modeled within a linear multivariate framework—the information transferred between two network nodes conditioning to the remaining nodes corresponds to the well-known measure of Granger causality (GC) formulated in a multivariate context [14], and the measures of redundancy and synergy can be obtained as separate measures through a so-called partial information decomposition (PID) [15].

The tools of information dynamics have contributed substantially to the development of the field of Network Physiology, with particular regard to the description of complex organ system interactions in various physiological states and conditions. In fact, measures information transfer and information modification have proven useful to the understanding of the dynamic interactions that are essential to produce different physiological states, e.g., wake and sleep [7,8,16,17], rest and physiological stress [18,19], relaxed conditions and mental workload [20,21], neutral states and emotion elicitation [22,23]. However, despite its growing appeal and widespread use in physiology and in diverse branches of science [24,25,26,27], the field of information dynamics is still under development and different aspects have to be further explored to fully exploit its potential. Recent developments have led to the formulation of a computational framework for the analysis of information dynamics which makes use of the state–space (SS) formulation of vector autoregressive models (VAR) and of the formation of reduced linear regression models [28,29] whose prediction error variance is related to the entropies needed for the computation of GC and PID measures [30]. The framework exhibits high computational reliability when compared with classical regression approaches for the estimation of Granger-causal measures [30], and is being increasingly used to assess information dynamics in the context of Network Physiology [3,19].

Nevertheless, being based entirely on linear parametric modeling, it suffers from the known vulnerability to the lack of data of the standard VAR identification techniques such as the Ordinary Least Square (OLS) or the Levison’s recursive algorithm for the solution of Yule-Walker equations. This issue exposes the identification process to increased bias and variance of the estimated parameters [31], and may result in ill-posed regression problems when the regressor’s matrix approaches singularity [32]. As pointed out in the literature, the ratio between the number of data samples available and the number of regression coefficients to be estimated should be at least equal to 10 to guarantee the accuracy of the estimation procedure [31,33,34]. This implies that the length of the time series used for VAR identification needs to increase proportionally with the number of processes jointly analyzed, which imposes a limitation to the size of the network that can be investigated if short datasets are available for the analysis. This is the case of common Network Physiology applications, where typically only short realizations of stationary multivariate physiological processes are available due to the different temporal scales and dynamics of the physiological signals involved.

To cope with the reduction of accuracy in the estimation process when dealing with a large number of time series and/or a small amount of data samples available, different strategies have been proposed in the literature such as the so-called partial conditioning [35] or the use of time-ordered restricted VAR models that are specifically built only for the computation of GC [36]. A former, more general solution is the use of penalized regression techniques that regularize a linear regression problem using one or more constraints [37]. Among them, the Least Absolute Shrinkage and Selection Operator (LASSO) uses a constraint based on the $l_{1}$ norm that if applied directly on the regression problem, yields to a sparse coefficients matrix which leads to a reduction of the mean square error in conditions of data paucity [38]. Penalized regression techniques implemented for GC analysis have been successfully applied in many different contexts, ranging from simulation studies [39] to the analysis of electroencephalographic signals [34,40,41], neuroimaging data [42] and Macroeconomic data [43]. In the present work, the LASSO regression is embedded in the VAR-SS framework for the computation of information dynamics, and is compared with the traditional OLS regression as regards its capability to estimate conditional information transfer and PID measures both in benchmark networks of simulated multivariate processes and in real networks of multiple physiological time series.

We show that it is possible, also in conditions of data paucity, to accurately reconstruct both the topology and the patterns of information transfer in networks of several coupled Gaussian systems exhibiting complex interactions, and to extract physiologically plausible patterns of interaction between the cardiovascular, respiratory and brain systems explored in healthy subjects during different conditions of mental stress elicited by sustained attention or mental arithmetic tasks [3,21,44].

The algorithms for the VAR-SS model identification based on the LASSO regression, with the subsequent computation of conditional information transfer and PID measures, are collected in the PID-LASSO MATLAB toolbox, which can be downloaded from http://github.com/YuriAntonacci/PID-LASSO-toolbox and http://lucafaes.net/PIDlasso.html (in Supplementary Materials).

## 2. Materials and Methods

### 2.1. Vector Autoregressive Model Identification

Let us consider a dynamical system Y, whose activity is mapped by a discrete-time stationary vector stochastic process composed of *M* real-valued zero-mean scalar processes, $Y=[Y_{1}\cdots Y_{M}]$. Considering the time step *n* as the current time, the present and the past of the vector stochastic process are denoted as $Y_{n}=\left[Y_{1,n}\cdots Y_{M,n}\right]$ and $Y_{n}^{-}=\left[Y_{n-1}Y_{n-2}\cdots \right]$, respectively. Moreover, assuming that Y is a Markov process of order *p*, its whole past history can be truncated using *p* time steps, i.e., using the Mp-dimensional vector $Y_{n}^{p}$ such that $Y_{n}^{-}\approx Y_{n}^{p}=\left[Y_{n-1}\cdots Y_{n-p}\right]$. Then, in the linear signal processing framework, the dynamics of *Y* can be completely described by the Vector autoregressive (VAR) model:

(1) $$Y_{n}=\sum_{k=1}^{p}Y_{n-k}A_{k}+U_{n},$$

where $A_{k}$ is an M×M matrix containing the autoregressive (AR) coefficients, and $U=[U_{1}\cdots U_{M}]$ is a vector of *M* zero-mean white processes, denoted as innovations, with M×M covariance matrix $\Sigma \equiv E[U_{n}^{T}U_{n}]$ (E is the expectation value).

Let us now consider a realization of the process Y involving *N* consecutive time steps, collected in the N×M data matrix $\left[y_{1};\cdots ;y_{N}\right]$, where the operator “;” stands for row separation, so that the $i^{th}$ row is a realization of $Y_{i}$, i.e., $y_{i}=\left[y_{1,i}\ldots y_{M,i}\right],i=1,\ldots ,N$, and the $j^{th}$ column is the time series collecting all realizations of $Y_{j}$, i.e., ${\left[y_{j,1}\ldots y_{j,N}\right]}^{T},j=1,\ldots ,M$,. The Ordinary Least Square (OLS) identification finds an optimal solution for the problem (1) by solving the following linear quadratic problem:

(2) $$\hat{A}={\mathrm{argmin}}_{A}||y-y^{p}A{\left|\right|}_{2}^{2},$$

where $y=[y_{p+1};\cdots ;y_{N}]$ is the (N−p)×M matrix of the predicted values, $y^{p}=\left[y_{p+1}^{p};\cdots ;y_{N}^{p}\right]$ is the (N−p)×Mp matrix of the regressors and $A=[A_{1};\cdots ;A_{p}]$ is the Mp×M coefficient matrix. The problem has a solution in a closed form $\hat{A}={\left({\left[y^{p}\right]}^{T}y^{p}\right)}^{-1}{\left[y^{p}\right]}^{T}y$ for which the residual sum of squares is minimized (RSS) [33,45]. When N−p≤Mp the OLS does not guarantee the uniqueness of the solution since the matrix $\left({\left[y^{p}\right]}^{T}y^{p}\right)$ becomes singular [34,45]. Even in this situation, it is possible to solve the problem stated in Equation (1) through the Least Absolute Shrinkage and Selection Operator (LASSO) which introduces a constraint in the linear quadratic problem (2) [37]:

(3) $$\hat{A}={\mathrm{argmin}}_{A}(||y-y^{p}{A||}_{2}^{2}+{\lambda ||A||}_{1}).$$

In Equation (3), the additional term based on the $l_{1}$ norm forces a sparse a solution such that some of the VAR coefficients are shrunk to zero, with the shrinkage parameter λ controlling the trade-off between the number of non-zero coefficients selected in the matrix $\hat{A}$ and the residual sum of squares (RSS). Even if the problem (3) admits a solution, it will not be in a closed form since the $l_{1}$ norm is not differentiable at zero [38]. The optimal value of λ for the solution of the problem (3) requires a cross-validation approach for its determination. Typically, a predefined interval of values for λ is defined such that the biggest value provides an estimated AR matrix of zeroes and the lowest provides a dense AR matrix [46] (in this work, 300 values of λ were selected). Subsequently, using an hold-out approach, as described in [47], it is possible to independently draw 90% of the observations of the predicted values and of the regressors (rows of y and $y^{p}$) as training set and keeping the remaining 10% for the testing set. Training and test sets are then reduced to zero mean and unit variance and, for each assigned λ, the number of non-zero coefficients is evaluated for the matrix $\hat{A}$ estimated from the training set, and the corresponding RSS is computed on the test set. After repeating this operation several times (10 in this work) by randomly changing the training and testing sets, the optimal value of λ is chosen as the one that minimizes the ratio between RSS and the number of non-zero VAR coefficients [48]. The matrix of AR coefficients $\hat{A}$ is then estimated by using the estimated optimal value of λ.

### 2.2. Measures of Information Transfer

Considering the overall observed process $Y=[Y_{1}\cdots Y_{M}]$, let us assume $Y_{j}$ as the target process and $Y_{i}$ as the source process, with the remaining M−2 processes collected in the vector $Y_{s}$ where s={1,…,M}\{i,j}. Then, the transfer entropy (TE) from $Y_{i}$ to $Y_{j}$ quantifies the amount of information that the past of the source, $Y_{i,n}^{p}$, provides about the present of the target, $Y_{j,n}$, over and above the information already provided by the past of the target itself, $Y_{j,n}^{p}$, and is defined as follows [2,49]:

(4) $$T_{i\to j}=I\left(Y_{j,n};Y_{i,n}^{p}|Y_{j,n}^{p}\right)=H\left(Y_{j,n}|Y_{j,n}^{p}\right)-H\left(Y_{j,n}|Y_{j,n}^{p},Y_{i,n}^{p}\right)$$

where I(·;·|·) represents the conditional mutual information and H(·|·) represents the conditional entropy [50]. In the presence of two sources $Y_{i}$ and $Y_{k}$, the information transferred towards the target $Y_{j}$ from the two sources taken together is quantified by the joint transfer entropy (jTE):

(5) $$T_{ik\to j}=I\left(Y_{j,n};Y_{i,n}^{p},Y_{k,n}^{p}|Y_{j,n}^{p}\right)=H\left(Y_{j,n}|Y_{j,n}^{p}\right)-H\left(Y_{j,n}|Y_{j,n}^{p},Y_{i,n}^{p},Y_{k,n}^{p}\right)$$

where $Y_{k,n}^{p}$ represents the past of the source *k*. Then, a possible way to decompose the jTE is that provided by the so-called partial information decomposition (PID). The PID expands the information transferred jointly from two sources to a target in four different quantities, reflecting the unique information transferred from each individual source to the target, measured by the unique TEs $U_{i\to j}$ and $U_{k\to j}$, and the redundant and synergistic information transferred from the two sources to the target, measured by the redundant TE $R_{ik\to j}$ and the synergistic TE $S_{ik\to j}$ [51]. These four measures are related to each other and to the joint and individual TEs from each source to the target by the following equations:

(6) $$T_{ik\to j}=U_{i\to j}+U_{k\to j}+R_{ik\to j}+S_{ik\to j},$$

(7) $$T_{i\to j}=U_{i\to j}+R_{ik\to j}$$

(8) $$T_{k\to j}=U_{k\to j}+R_{ik\to j}$$

In the PID defined above, the terms $U_{i\to j}$ and $U_{k\to j}$ quantify the parts of the information transferred to the target process $Y_{j}$ which are unique to the source processes $Y_{i}$ and $Y_{k}$, respectively, mirroring the contributions to the predictability of the target that can be obtained from one of the sources but not from the other. Each of these unique contributions sums up with the redundant TE to retrieve the information transfer defined by the classical measure of the bivariate TE, thus indicating that $R_{ik\to j}$ pertains to the part of the information transferred individually, yet redundantly from a source to the target. The term $S_{ik\to j}$ refers to the synergy between the two sources while they transfer information to the target, intended as the information that is uniquely obtained taking the two sources $Y_{i}$ and $Y_{k}$ together, but not considering them alone. While several implementations of the PID exists depending on how a fourth equation is formulated to complete the definitions (6-8), in the case of joint Gaussian processes it has been shown that an unifying formulation is that defining the redundant transfer as the minimum information transferred individually by each source to the target, i.e., $R_{ik\to j}=min\left(T_{i\to j},T_{k\to j}\right)$ [15].

In addition to the measures defining the PID, another important information measure used to detect the topological structure of direct interactions in a network of *M* interacting processes is the conditional transfer entropy (cTE). With the notation introduced above for the overall vector process Y, the cTE from a driver process $Y_{i}$ to a target process $Y_{j}$ computed considering the other processes in the network collected in $Y_{s}$, is defined as:

(9) $$T_{i\to j|s}=I\left(Y_{j,n};Y_{i,n}^{p}|Y_{j,n}^{p},Y_{s,n}^{p}\right)=H\left(Y_{j,n}|Y_{j,n}^{p},Y_{s,n}^{p}\right)-H\left(Y_{j,n}|Y_{n}^{p}\right)$$

The cTE quantifies the amount of information contained in the present state of the target process that can be predicted by the past states of the source process, above and beyond the information that is predicted already by the past states of the target and of the all other processes [14]. An implication of this definition is that non-zero values of the cTE $T_{i\to j|s}$ correspond to the presence of a direct causal interaction from $Y_{i}$ to $Y_{j}$, which is typically depicted, in a network representation where nodes are associated with processes and edges with significant causal interactions, with an arrow connecting the $i^{th}$ and $j^{th}$ nodes.

### 2.3. Computation of the Measures of Information Transfer for Multivariate Gaussian Processes

When the observed multivariate process Y has a joint Gaussian distribution, the information- theoretic measures described in Section 2.2 can be formulated in an exact way based on the linear VAR representation provided in Section 2.1. Indeed, it has been shown that the covariance matrices of the observed vector process and of the residuals of the formulation (1) contain, in the case of jointly distributed Gaussian processes, all of the entropy differences which are needed to compute the information transfer [52]. In turn, these entropy differences are expressed by the concept of partial covariance formulated in the context of linear regression analysis. Specifically, defining $E_{j|j,n}=Y_{j,n}-E\left[Y_{j,n}|Y_{j,n}^{p}\right]$ and $E_{j|ij,n}=Y_{j,n}-E\left[Y_{j,n}|Y_{i,n}^{p},Y_{j,n}^{p}\right]$ as the prediction errors of a linear regression of $Y_{j,n}$ performed respectively on $Y_{j,n}^{p}$ and $\left[Y_{i,n}^{p}Y_{j,n}^{p}\right]$, the conditional entropies $H(Y_{j,n}|Y_{j,n}^{p})$ and $H(Y_{j,n}|Y_{j,n}^{p},Y_{i,n}^{p})$ can be expressed as functions of the prediction error variances $\lambda_{j|j}=E\left[E_{j|j,n}^{2}\right]$ and $\lambda_{j|ij}=E\left[E_{j|ij,n}^{2}\right]$ as follows [14,53]:

(10a) $$H\left(Y_{j,n}|Y_{j,n}^{p}\right)=\frac{1}{2}ln2\pi e\lambda_{j|j},$$

(10b) $$H\left(Y_{j,n}|Y_{j,n}^{p},Y_{i,n}^{p}\right)=\frac{1}{2}ln2\pi e\lambda_{j|ij},$$

from which the TE from $Y_{i}$ to $Y_{j}$ can be retrieved using (7):

(11) $$T_{i\to j}=\frac{1}{2}ln\frac{\lambda_{j|j}}{\lambda_{j|ij}}.$$

Following similar reasoning, the jTE from $\left(Y_{i},Y_{k}\right)$ to $Y_{j}$ can be defined as:

(12) $$T_{ik\to j}=\frac{1}{2}ln\frac{\lambda_{j|j}}{\lambda_{j|ijk}},$$

where $\lambda_{j|ijk}=E\left[E_{j|ijk,n}^{2}\right]$ is the variance of the prediction error of a linear regression of $Y_{j,n}$ on $\left(Y_{i,n}^{p},Y_{j,n}^{p},Y_{k,n}^{p}\right)$ with prediction error $E_{j|ijk,n}=Y_{j,n}-E\left[Y_{j,n}|Y_{i,n}^{p},Y_{j,n}^{p},Y_{s,n}^{p}\right]$, and the cTE from $Y_{i}$ to $Y_{j}$ given $Y_{s}$ can be defined as:

(13) $$T_{i\to j|s}=\frac{1}{2}ln\frac{\lambda_{j|js}}{\lambda_{j|ijs}},$$

where $\lambda_{j|js}=E\left[E_{j|js,n}^{2}\right]$ is the variance of the prediction error of a linear regression of $Y_{j,n}$ on $\left(Y_{j,n}^{p},Y_{s,n}^{p}\right)$ with prediction error $E_{j|js,n}=Y_{j,n}-E\left[Y_{j,n}|Y_{j,n}^{p},Y_{s,n}^{p}\right]$ and $\lambda_{j|ijs}=E\left[E_{j|ijs,n}^{2}\right]$ is the variance of the prediction error of a linear regression of $Y_{j,n}$ on $Y_{n}^{p}$ with prediction error $E_{j|ijs,n}=Y_{j,n}-E\left[Y_{j,n}|Y_{n}^{p}\right]$. Moreover, from the definitions in Section 2.2 it is then possible to obtain the redundant TE, the synergistic TE and the unique TEs in addition to the cTE. Therefore, the computation of all the information measures amounts to calculate the partial variances to be inserted in Equations (11)–(13). In the following subsection we report how to derive such partial variances exploiting the State–Space formulation of the VAR model (1) [30].

#### Formulation of State–Space Models

A discrete state–space (SS) model is a linear model in which a set of input, output and state variables are related by first order difference equations [29]. The VAR model (1) can be represented equivalently as an SS model ([54]) which relates the observed process Y to an unobserved state process Z through the observation equation

(14) $$Y_{n}=CZ_{n}+E_{n},$$

and describes the update of the state process through the state equation

(15) $$Z_{n+1}=AZ_{n}+KE_{n}.$$

The innovations $E_{n}$ of Equations (14) and (15) are equivalent to the innovations $U_{n}$ in (1) and thus have covariance matrix $\Phi \equiv E[E_{n}^{T}E_{n}]=\Sigma$. This representation, typically denoted as “innovation form” SS model (ISS), also demonstrates the Kalman Gain matrix **K**, the state matrix **A** and the observation matrix **C**, which can all be computed from the original VAR parameters in (1) as reported in ([54]). Starting from the parameters of an ISS model is possible to compute any partial variance $\lambda_{j|a}$, where the subscript *a* denotes any combination of indexes ∈(1,…,M), by evaluating the innovation of a “submodel” obtained removing from the observation Equation (14) the variables not included in *a*. Furthermore, in this formulation the state Equation (15) remains unaltered and the observation equation of relevant submodel becomes:

(16) $$Y_{n}^{\left(a\right)}=C^{\left(a\right)}Z_{n}+E_{n}^{\left(a\right)},$$

where the subscript ${}^{a}$ denotes the selection of the rows with indices *a* of a vector or a matrix. As demonstrated in [28,30], the submodel (15) and (16) is not in ISS form, but can be converted into ISS by solving a Discrete Algebraic Riccati equation (DARE). Then, the covariance matrix of the innovations $\Phi^{\left(a\right)}=E\left[E_{n}^{(a)T}E_{n}^{\left(a\right)}\right]$ includes the desired error variance $\lambda_{j|a}$ as diagonal element corresponding to the position of the target $Y_{j}$. Thus, it is possible to compute all the partial variances needed for the evaluation of all the information measures introduced, starting from a set of ISS parameters. In particular, these parameters can be directly extracted by the knowledge of the parameters of the original VAR model (i.e., $A_{1},\ldots ,A_{p},\Sigma$), which in this study are estimated by identifying the VAR model (1) making use of either the OLS method or the LASSO regression.

### 2.4. Testing the Significance of the Conditional Transfer Entropy

Since the cTE $T_{i\to j|s}$ is a measure of the information transferred directly (i.e., without following indirect paths) from the source $Y_{i}$ to the target $Y_{j}$, and for Gaussian processes is equivalent to conditional Granger causality [14], it is of interest to perform the assessment of its statistical significance with the aim to establish the existence of a direct link from the $i^{th}$ node to the $j^{th}$ node of the observed network of interacting processes. In this work, the significance of cTE, computed after OLS identification of the VAR model, was tested generating sets of surrogate time series which share the same power spectrum of the original time series but are otherwise uncorrelated. Specifically, 100 sets of surrogate time series were generated using the Iterative Amplitude Adjusted Fourier Transform (IAAFT) procedure [55]; then, the cTE was estimated for each surrogate set, a threshold equal to the 95**^th^** percentile of its distribution on the surrogates was determined for each directed link, and the link was detected as statistically significant when the original cTE was above the threshold. In the case of LASSO, the statistical significance of the estimated cTE values was determined exploiting the sparseness of the identification procedure. Since LASSO model identification always produces a sparse matrix with several VAR coefficients equal to zero, the cTE values result exactly zero when the coefficients along the investigated direction are zero at each time lag; on the contrary, cTE is positive, and was considered to be statistically significant in this study, when at least one coefficient is non-zero along the considered direction.

## 3. Simulation Experiments

This section reports two simulation studies performing a systematic evaluation of the performances of the two VAR identification methodologies (OLS and LASSO) employed for the practical computation of the measures of information transfer in known networks assessed with different amount of data samples available. First, we study the behavior of the measures of information transfer and information modification in a four-variate VAR process specifically configured to reproduce coexisting forms of redundant and synergistic interactions between source processes sending information towards a target [15,30]. Second, with specific focus on the estimation of the cTE and of its statistical significance, we compared the ability of OLS and LASSO to reconstruct an assigned network topology in a ten-variate VAR process exhibiting a random interaction structure with fixed density of connected nodes [34,56]

### 3.1. Simulation Study I

#### 3.1.1. Simulation Design and Realization

**Figure 1:**
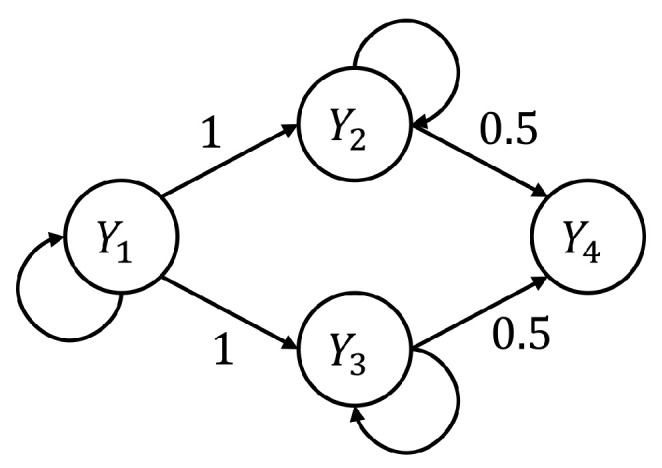
Graphical representation of the four-variate VAR (Vector Autoregressive) process realized in the first simulation according to Equation (17). Network nodes represent the four simulated processes, and arrows represent the imposed causal interactions (self-loops depict influences from the past to the present sample of a process).

Simulated multivariate time series (*M*=4) were generated as realizations of the following VAR(2) process depicted in Figure 1 [2,30,57]:

(17a) $$Y_{1,n}=2\rho_{1}\cos\left(2\pi f_{1}\right)Y_{1,n-1}-\rho_{1}^{2}Y_{1,n-2}+U_{1,n},$$

(17b) $$Y_{2,n}=2\rho_{2}\cos\left(2\pi f_{2}\right)Y_{2,n-1}-\rho_{2}^{2}Y_{2,n-2}+Y_{1,n-1}+U_{2,n},$$

(17c) $$Y_{3,n}=2\rho_{3}\cos\left(2\pi f_{3}\right)Y_{3,n-1}-\rho_{3}^{2}Y_{3,n-2}+Y_{1,n-1}+U_{3,n},$$

(17d) $$Y_{4,n}=\frac{1}{2}Y_{2,n-1}+\frac{1}{2}Y_{3,n-1}+U_{4,n},$$

In (17), $U=[U_{1}\ldots U_{4}]$ is a vector of zero-mean uncorrelated white noises with unit variance (i.e., with covariance Σ≡I). The VAR parameters are selected to allow autonomous oscillations for $Y_{1},Y_{2},$ and $Y_{3}$ by placing, in the VAR representation in the Z−domain, complex-conjugate poles with modulus $\rho_{i}$ and phase $2\pi f_{i},i=1,2,3$; here we set pole modulus $\rho_{1}=\rho_{2}=\rho_{3}=0.95$ and pole frequency $f_{1}=0.1$, $f_{2}=f_{3}=0.25$. Moreover, interactions between different processes were set to allow a common driver effect $y_{2}\leftarrow y_{1}\to y_{3}$ and unidirectional couplings $y_{2}\to y_{4}$ and $y_{3}\to y_{4}$, with weights indicated in Figure 1. With these settings, 100 realizations of the processes were generated under different values of the parameter *K* defined as the ratio between the number of data samples available (*N*) and the number of AR coefficients to be estimated (Mp); the parameter *K* was varied in the range (1,2,5,10,30), so that the length of the simulated time series was N=8 when K=1 and N=240 were when K=30. For each realization and for each value of *K*, all the measures appearing in the PID of the information transfer were computed by exploiting the SS approach applied to the VAR parameters estimated through OLS or LASSO identification; PID analysis was performed considering either $Y_{4}$ or $Y_{1}$ as the target process, and both $Y_{2}$ and $Y_{3}$ as the source processes. Then, the bias and variance of each estimated PID measure were assessed, for each *K* and separately for OLS and LASSO, respectively as the absolute difference between the mean value of the measure over the 100 realizations and its theoretical value computed using the true values imposed for the VAR parameters, and as the sample variance estimated over the 100 realizations.

#### 3.1.2. Simulation Results

**Figure 2:**
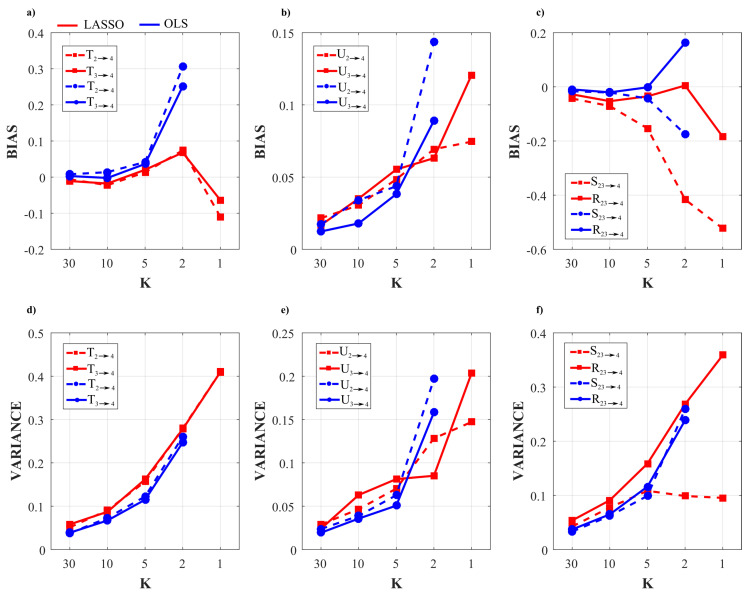
Accuracy of PID (Partial Information Decomposition) measures computed for the VAR processes of Simulation I when $Y_{4}$ is taken as the target process. Panels report the bias (**a**–**c**) and the variance (**d**–**f**) relevant the computation of the TE (Transfer Entropy) from $Y_{2}$ to $Y_{4}$ and from $Y_{3}$ to $Y_{4}$ (**a**,**d**), the unique TE from $Y_{2}$ to $Y_{4}$ and from $Y_{3}$ to $Y_{4}$ (**b**,**e**) and the redundant and synergistic TE from $Y_{2}$ and $Y_{3}$ to $Y_{4}$ (**c**,**f**).

**Figure 3:**
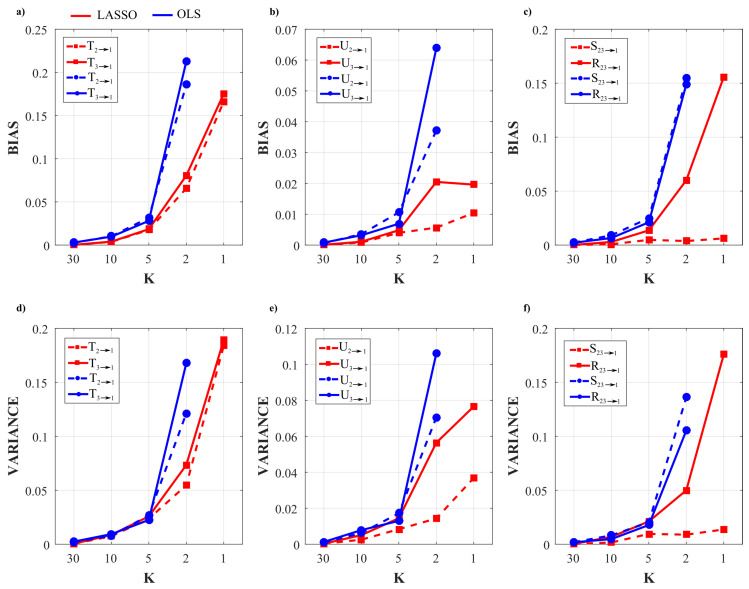
Accuracy of PID measures computed for the VAR processes of Simulation I when $Y_{1}$ is taken as the target process. Panels report the bias (**a**–**c**) and the variance (**d**–**f**) relevant the computation of the TE from $Y_{2}$ to $Y_{1}$ and from $Y_{3}$ to $Y_{1}$ (**a**,**d**), the unique TE from $Y_{2}$ to $Y_{1}$ and from $Y_{3}$ to $Y_{1}$ (**b**,**e**) and the redundant and synergistic TE from $Y_{2}$ and $Y_{3}$ to $Y_{1}$ (**c**,**f**).

Figure 2 and Figure 3 show the trends of bias and variance associated with the estimation of TE ($T_{2\to j},T_{3\to j}$), redundant TE ($R_{23\to j}$), synergistic TE ($S_{23\to j}$) and unique TEs ($U_{2\to j}$, $U_{3\to j}$) respectively when j=4 (target process $Y_{4}$) and j=1 (target process $Y_{1}$), computed after VAR model identification using OLS (blue) and LASSO (red) and depicted as a function of the ratio *K* between time series length and number of model parameters.

As a general result, both figures show that the accuracy of all estimates of the PID measures is strongly influenced by the amount of data available, with a progressive increase of both the bias and the variance of the estimates with the decrease of the parameter *K*. The LASSO regression exhibits a substantially better performance in the estimation of the PID measures particularly when the amount of data samples is scarce (K≤2). In the most challenging condition of K=1 (number of AR coefficients equal to the number of data points) the results are reported only for the LASSO regression since in this condition for OLS it was impossible to evaluate the PID measures due to the non-convergence of the DARE equation solution during the computation. In the other cases (K∈(5,10,30)) the two identification methods show comparable trends, with slightly better performance exhibited by OLS identification in the assessment of non-zero PID measures (Figure 2), and by LASSO identification in the assessment of zero PID measures (Figure 3).

In fact, when $Y_{4}$ is taken as target process, the sources $Y_{2}$ and $Y_{3}$ send the same amount information towards the target and this information is entirely redundant ($T_{2\to 4}=T_{3\to 4}=R_{23\to 4}=0.63,U_{2\to 4}=U_{3\to 4}=0$); moreover, a non-negligible amount of synergistic information transfer is present ($S_{23\to 4}=0.56$) [30]. As reported in Figure 2, the estimates of the non-zero quantities ($T_{2\to 4},T_{3\to 4},R_{23\to 4},S_{23\to 4}$) assessed through LASSO-VAR identification exhibit higher variance than those assessed through the OLS, as well a slight negative bias which becomes relevant only in the case of the synergistic TE; in such a case the underestimation of $S_{23\to 4}$ is present also after OLS identification when K=2 (Figure 2c).

When the process $Y_{1}$ is taken as the target, all the PID measures are null ($T_{2\to 1}=T_{3\to 1}=U_{2\to 1}=U_{3\to 1}=S_{23\to 1}=R_{23\to 1}=0$) because no causal interactions are directed towards $Y_{1}$. As shown in Figure 3, in this case the LASSO identification outperforms the OLS method, showing lower bias and variance for all values of *K* with evident improvement in the performance when K≤2. Interestingly, for low values of *K* the LASSO regression detected the absence of synergy with more accuracy than that of redundancy (Figure 3c,f).

### 3.2. Simulation Study II

#### 3.2.1. Simulation Design and Realization

**Figure 4:**
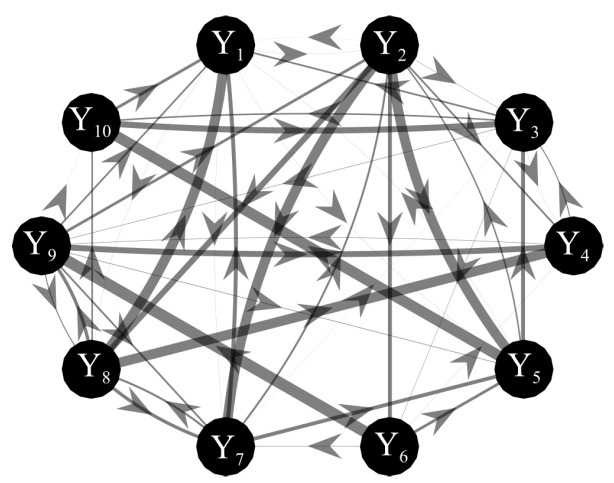
Graphical representation for one of the ground-truth networks of Simulation II. Arrows represent the existence of a link, randomly assigned, between two nodes in the network. The thickness of the arrows is proportional to the strength of the connection, with a maximum value for the cTE equal to 0.15. The number of connections for each network is set to 45 out of 90.

Simulated multivariate time series (M=10) were generated as realizations of a VAR(10) model fed by white Gaussian noises with variance equal to 0.1. The simulated networks have a ground-truth structure with a density of connected nodes equal to 50% in which non-zero AR parameters were set assigning randomly the lag in the range (1–10) and the coefficient value in the interval [−0.6,0.6] [58]. A representative example of one possible generated network is shown in Figure 4, where the strength of the directed links is provided by the theoretical cTE computed between two processes starting from the true AR parameters. Under these constraints, 100 realizations (each with its specific network structure) of the VAR(10) process were generated with different values of the parameter *K* in the range (1,2,5,10,30), so that the length of the simulated time series was N=100 when K=1 and N=3000 were when K=30. For each realization and for each value of *K*, the cTE between each pair of processes was computed by exploiting the SS approach applied to the VAR parameters estimated through OLS or LASSO identification. Then, the bias and variance of the cTE estimates obtained through OLS and LASSO identification were assessed separately for the connections with zero and non-zero cTE as explained in the following subsection.

#### 3.2.2. Performance Evaluation

The performances of LASSO and OLS were assessed both in terms of the accuracy in estimating the strength of the network links through the absolute values of the cTE measure, and in terms of the ability to reconstruct the network structure through the assessment of the statistical significance of cTE. The first analysis was performed separately for non-null and null links computing the bias of cTE through the comparison between the estimated and theoretical cTE values. Specifically, for each pair of network nodes represented by the processes $Y_{i}$ and $Y_{j}$, the theoretical cTE obtained from the true VAR parameters, $T_{i\to j|s}$, was compared with the corresponding estimated cTE value, ${\hat{T}}_{i\to j|s}$, using a measure of absolute bias (bias) if the theoretical link is null, and a normalized measure of bias ($bias_{N}$) if the theoretical link is non-null [59]:

(18a) $$bias=|T_{i\to j|s}-{\hat{T}}_{i\to j|s}|,$$

(18b) $$bias_{N}=|\frac{T_{i\to j|s}-{\hat{T}}_{i\to j|s}}{T_{i\to j|s}}|.$$

Then, for each network, the values of bias and $bias_{N}$ were averaged respectively across the 45 non-null links and across the 45 null links to get individual measures, denoted as BIAS and $BIAS_{N}$. Finally, the distributions of BIAS and $BIAS_{N}$ were assessed across the 100 simulated network structures and presented separately for OLS and LASSO.

Second, the ability of OLS and LASSO to detect the absence or presence of network links based on the statistical significance of the cTE was tested comparing the two adjacency matrices representative of the estimated and theoretical network structures. This can be seen as a binary classification task where the existence (class 1) or absence (class 0) of each estimated connection is assessed (using surrogate data for OLS and looking for zero/non-zero estimated coefficients for LASSO) and compared with the underlying ground-truth structure. Performances were assessed through the computation of the false positive rate (FPR, measuring the fraction of null links for which a statistically significant cTE was detected), false negative rate (FNR, measuring the fraction of non-null links for which the cTE was detected as non-significant) and accuracy (ACC, measuring the fraction of false detections) parameters [40,60]. Each of these performance measures was obtained across the network links for each individual network, and its distribution across the 100 simulated network structures was then presented separately for OLS and LASSO.

#### 3.2.3. Statistical Analysis

For this simulation study, five different repeated measures two-way ANOVA tests, one for each performance parameter (BIAS,$BIAS_{N}$,FNR,FPR,ACC) were performed, to evaluate the effects of different values of K (varied in the range [30,10,5,2]) and different identification methodologies ([OLS,LASSO]) on performance parameters.

The Greenhouse–Geisser correction for the violation of the spherical hypothesis was used in all analyses. The Tukey’s post-hoc test was used for testing the differences between sub-levels of ANOVA factors. The Bonferroni-Holm correction was applied for multiple ANOVAs computed on different performance parameters.

#### 3.2.4. Results of the Simulation Study

The results of the two-way repeated measures ANOVAs, expressed in terms of F-values and computed separately on all the performance parameters considering K and TYPE (identification method used) as main factors, are reported in Table 1.

The two-way ANOVAs reveal a strong statistical influence of the main factors K and TYPE and of their interaction on all the performance parameters analyzed. It is worth of note that the level K=1 was not considered in the statistical analysis due to the non-convergence of the DARE equation for the OLS case.

**Figure 5:**
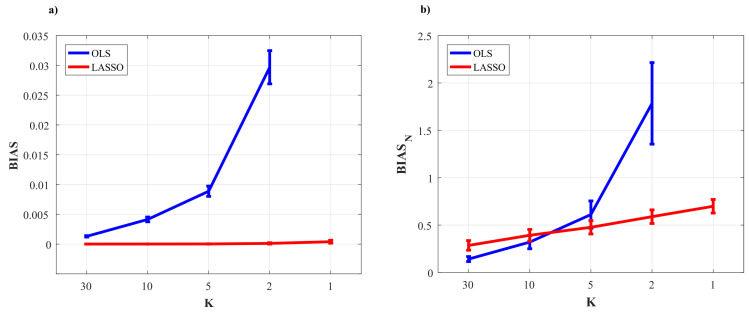
Distribution of the bias parameters computed for the null links (BIAS, **a**) and for the non-null links ($BIAS_{N}$, **b**) considering the interaction factor K × TYPE, expressed as mean value and 95% confidence interval of the parameter computed across 100 realizations of simulation II for OLS (blue line) and LASSO (red line) for different values of K.

Figure 5 reports the distribution of the parameters BIAS and $BIAS_{N}$ according to the interaction factor K × TYPE.

The comparison of the two VAR identification procedures shows that the trends for LASSO (red line) and OLS (blue line) are very different. In the analysis of the error committed in the estimation of the null links (parameter BIAS) the error of LASSO estimates is almost zero for all levels of K (even for K≤2 that are the most challenging situations), while OLS estimates show a sharp increase of the error with the decrease of data samples available for the estimation of cTE (Figure 5a). The analysis of the error committed in the estimation of the non-null links (parameter $BIAS_{N}$, Figure 5b) highlights that for both methods the error increases with decreasing the value of K. The two identification methods exhibit different performance as a function of the number of data samples available for the estimation procedure: when such number is high (K=30), the OLS assumes a significantly smaller bias than LASSO; when 10≤K≤5 there are no significant differences between the two methods; in the most challenging conditions with K<5 OLS exhibits a drastic rise of $BIAS_{N}$ towards 2 (which means an overestimation up to 200%), while LASSO identification allows limitation of the bias which remains below 1 even when K=1.

**Figure 6:**
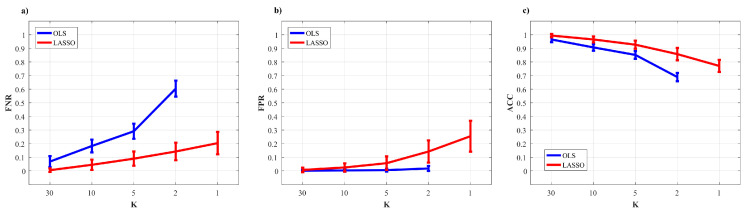
Distributions of FNR (**a**), FPR (**b**) and ACC (**c**) parameters considering the interaction factor K x TYPE, expressed as mean value and 95% confidence interval of the parameter computed across 100 realizations of simulation II for OLS (blue line) and LASSO (red line) for different values of K.

Figure 6 reports the distributions of the parameters FPR, FNR and ACC according to the interaction K × TYPE. The analysis of the rate of false negatives (Figure 6a) shows that the number of links incorrectly classified as null increases while decreasing the amount of data available (*K* decreasing from 10 to 2), with values of FNR rising from about 0.1 to about 0.6 using the OLS, and remaining much lower (between 0 and 0.2) using LASSO identification. On the other hand, the analysis of the rate of false positives (Figure 6b) returns opposite trends, with several absent links incorrectly classified as non-null which is stable and almost negligible using OLS, and exhibits a slight growth that leads the FPR value from 0 with K = 30 to about 0.25 for K = 1. The overall performance assessed through the ACC parameter is better using LASSO identification (Figure 6c): the rate of correctly detected links is comparable in the favorable condition K=30, while when K≤10 LASSO shows better performance (significantly higher values of ACC) than OLS and can reconstruct the network structure with a very good accuracy (∼80%) even in the challenging condition of K=1.

## 4. Application to Physiological Time Series

This section reports the application of the measures of information transfer, based on VAR models, estimated through OLS or LASSO identification, to a dataset of physiological time series previously collected with the aim of studying organ system interactions during different levels of mental stress [3]. The physiological time series measured for each subject were considered to be a realization of a vector stochastic process descriptive of the behavior of a composite dynamical system which forms a network of physiological interactions. Such network is composed of two distinct sub-networks, which are in turn formed by three nodes ("body" or peripheral sub-network) and four nodes (brain sub-network). The dynamic activity at each network node is quantified by a scalar process, as specifically defined in the next subsection.

### 4.1. Data Acquisition and Pre-Processing

Eighteen healthy participants with an age between 18 and 30 years were recorded during three different tasks inducing different levels of mental stress: a resting condition induced watching a relaxing video (R); a condition of mental stress induced by the execution of a mental arithmetic task (M) using an online tool in which the participants had to perform sums and subtractions of 3-digit numbers and write the solution in a text-box using the keyboard; a condition of sustained attention induced playing a serious game (G) which consisted of following a point moving on the screen using the mouse and trying to avoid different obstacles. All participants provided written informed consent. The experiment was approved by the Ethics Committees of the University of Trento. The study was in accordance with the Declaration of Helsinki.

The acquired physiological signals were the Electrocardiogram (ECG) signal, the respiratory signal (RESP) measured monitoring abdominal movements, the blood volume pulse (BVP) signal measured through a photoplethysmographic technique, and 14 Electroencephalogram (EEG) signals recorded at different locations in the scalp. After a pre-processing step performed in MatLab R2016b (Mathworks, Natick, MA, USA), seven physiological time series, each consisting of 300 data points and taken as a realization of the stochastic process representing the activity of specific physiological (sub)systems, were extracted from the recorded signals as follows: (1) the R-R tachogram, represented by the sequence of the time distances between consecutive R peaks of the ECG (process η); (2) The series of respiratory amplitude values, sampled at the onset of each detected R-R interval (process ρ): (3) the pulse arrival time (process π) obtained computing the time elapsed between each R peak in the ECG and the corresponding point of maximum derivative in BVP signal; the sequences of the EEG power spectral density, measured in consecutive time windows (lasting 2 s with 1 s overlap) of the EEG signal acquired at the electrode $F_{z}$, integrated within the bands 0.5−3Hz (process δ), 3−8Hz (process θ), 8−12Hz (process α), and 12−25Hz (process β). Before VAR modeling, the time series were reduced to zero mean and unit variance and checked for a restricted form of weak sense stationarity using the algorithm proposed in [61], which divides each time series into a given number of randomly selected sub-windows, assessing for each of them the stationarity of mean and variance. A detailed description of signal recording, experimental protocol and time series extraction can be found in [3,21].

### 4.2. Information Transfer Analysis

The seven time series obtained from each subject and from each condition were interpreted as a realization of a VAR process whose parameters $A_{1},\ldots ,A_{p},\Sigma$ were estimated with the two different identification methods under analysis (i.e., OLS and LASSO). The model order *p* was estimated, for each experimental condition and for each subject, using the Bayesian Information Criterion [62]. Then, two different analyses were performed through the application of the SS approach:

1. First, a PID analysis was performed for OLS and LASSO through the computation of the joint information transfer $T_{ik\to j}$ and the terms of its decomposition $U_{i\to j}$, $U_{k\to j}$, $R_{ik\to j}$, $S_{ik\to j}$. The analysis was performed collecting in the first source (index *i*) the processes [η,ρ,π] forming the so-called “body” sub-network that accounts for cardiac, cardiovascular and respiratory dynamics, and in the second source (index *k*) the processes [δ,θ,α,β] forming the “brain” sub-network that accounts for the different brain wave amplitudes; the analysis was repeated considering each one of the seven processes as the target process (j=[η,ρ,π,δ,θ,α,β]) and excluding it from the set of sources.
2. Second, the topological structure of the network of physiological interactions was detected computing the conditional transfer entropy $T_{i\to j|s}$ based on the two VAR identification methods combined with their method for assessing the statistical significance of cTE (i.e., using surrogate data for OLS and exploiting the intrinsic sparseness for LASSO). The analysis was performed between each pair of processes as driver and target (i,j=[η,ρ,π,δ,θ,α,β],i≠j) and collecting the remaining five processes in the conditioning vector with index *s*. As a quantitative descriptor of the network was used the in-strength, defined as the sum of all weighted inward links connected to one node [63]. Moreover, to describe the overall brain–body interactions the in-strength of the body sub-network due to brain sub-network (and vice-versa) was computed considering as link weights the percentage of subjects showing at least one statistically significant brain-to-body connection (and vice-versa). To study the involvement of each specific node in the network, the in-strength of each node was computed considering as link weights the cTE values of all network links pointing into the considered node.

### 4.3. Statistical Analysis

The effect of the different experimental conditions (R,M,G) on each PID measure computed for each target process (j=[η,ρ,π,δ,θ,α,β]) and for each VAR identification method (OLS, LASSO) was assessed with a Kruskal-Wallis test followed by a Wilcoxon rank sum test to assess statistical differences between pairs of conditions. Moreover, the Wilcoxon rank sum test was performed also to assess statistical differences between the two unique TEs ($U_{i\to j}$,$U_{k\to j}$) or between the redundant and synergistic TEs ($R_{ik\to j}$,$S_{ik\to j}$) assessed for a given experimental condition and for a given target process and identification method. Finally, in order to assess the effect of the experimental condition on the in-strength evaluated for each node in the network, a Kruskal-Wallis test was performed, followed by the Wilcoxon rank sum test between pairs of conditions.

### 4.4. Results of Real Data Application

**Figure 7:**
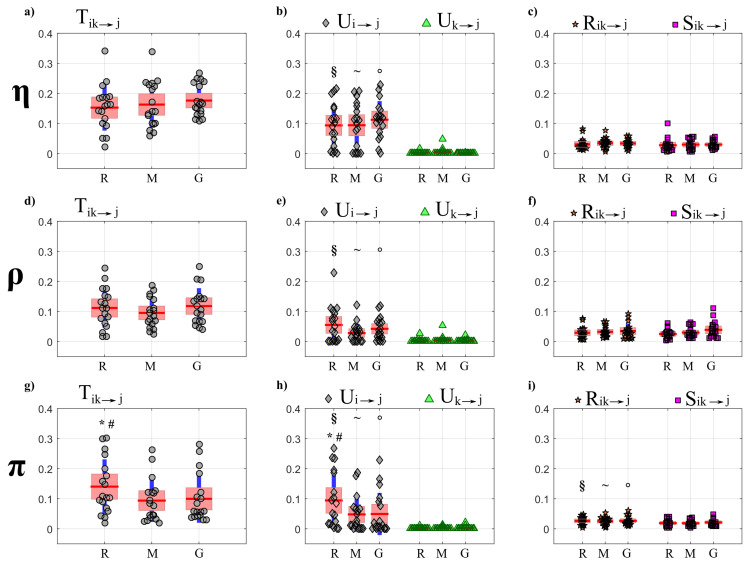
Partial Information Decomposition of brain–body interactions directed to the body nodes of the physiological network, assessed using OLS VAR identification. Box plots report the distributions across subjects (median: red lines; interquartile range: box; 10**^th^**–90**^th^** percentiles: blue lines) as well as the individual values (circles or triangles) of the PID measures (**a**,**d**,**g**: joint information transfer; **b**,**e**,**h**: unique information transfer; **c**,**f**,**i**: synergistic and redundant transfer) computed at rest (R), during mental stress (M) and during serious game (G) considering the RR interval (η), the respiratory amplitude (ρ), or the pulse arrival time (π) as the target process *j*, and the body and brain sub-networks as source processes *i* and *k*. Statistically significant differences between pairs of distributions are marked with * (R vs. M), with # (R vs. G), with *§* (R vs. R), with ∼ (M vs. M) and with ∘ (G vs. G).

**Figure 8:**
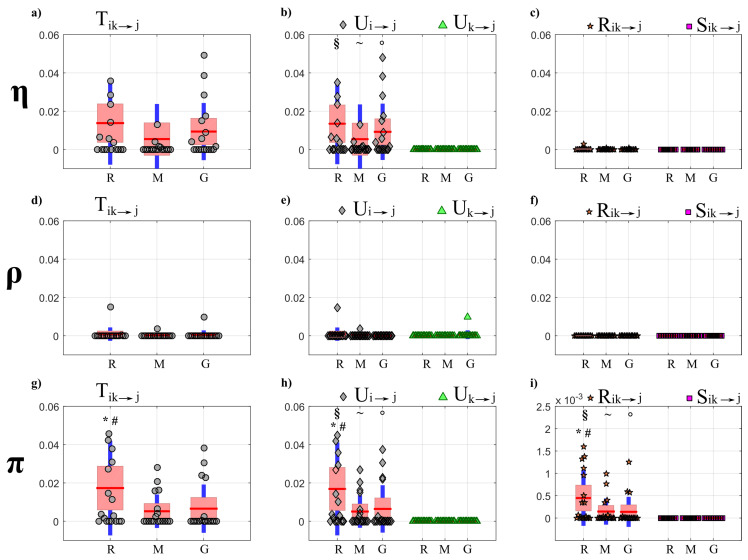
Partial Information Decomposition of brain–body interactions directed to the body nodes of the physiological network, assessed using LASSO-VAR identification. Box plots report the distributions across subjects (median: red lines; interquartile range: box; 10*^th^*–90*^th^* percentiles: blue lines) as well as the individual values (circles or triangles) of the PID measures (**a**,**d**,**g**: joint information transfer; **b**,**e**,**h**: unique information transfer; **c**,**f**,**i**: synergistic and redundant transfer) computed at rest (R), during mental stress (M) and during serious game (G) considering the RR interval (η), the respiratory amplitude (ρ), or the pulse arrival time (π) as the target process *j*, and the body and brain sub-networks as source processes *i* and *k*. Statistically significant differences between pairs of distributions are marked with * (R vs. M), with # (R vs. G), with *§* (R vs. R), with ∼ (M vs. M) and with ∘ (G vs. G).

**Figure 9:**
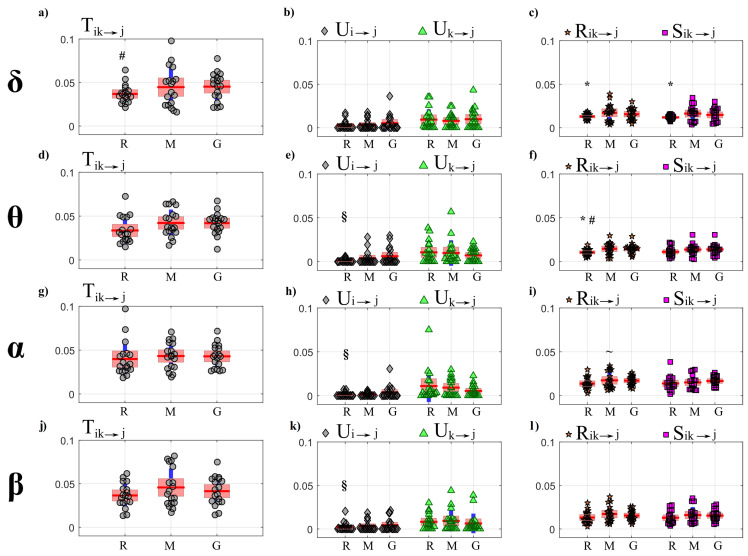
Partial Information Decomposition of brain–body interactions directed to the brain nodes of the physiological network, assessed using OLS VAR identification. Box plots report the distributions across subjects (median: red lines; interquartile range: box; 10*^th^*–90*^th^* percentiles: blue lines) as well as the individual values (circles or triangles) of the PID measures (**a**,**d**,**g**,**j**: joint information transfer; **b**,**e**,**h**,**k**: unique information transfer; **c**,**f**,**i**,**l**: synergistic and redundant transfer) computed at rest (R), during mental stress (M) and during serious game (G) considering the δ, θ, α, or β brain wave amplitude as the target process *j*, and the body and brain sub-networks as source processes *i* and *k*. Statistically significant differences between pairs of distributions are marked with * (R vs. M), with # (R vs. G), with *§* (R vs. R), with ∼ (M vs. M) and with ∘ (G vs. G).

**Figure 10:**
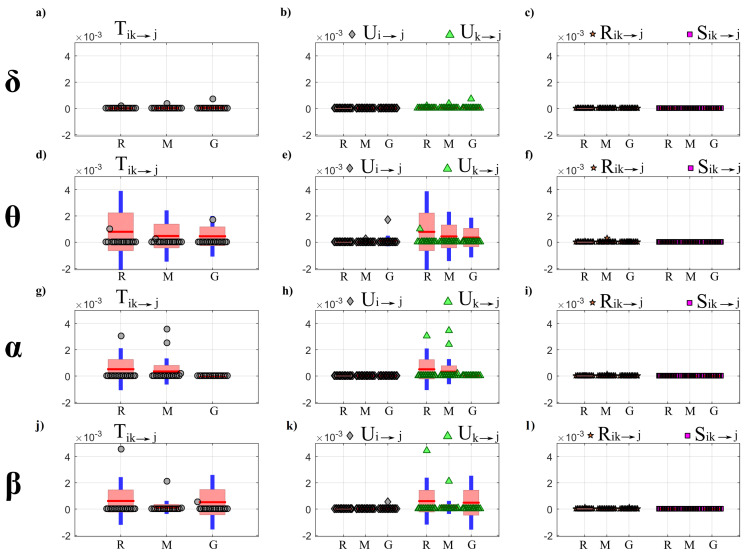
Partial Information Decomposition of brain–body interactions directed to the brain nodes of the physiological network, assessed using LASSO-VAR identification. Box plots report the distributions across subjects (median: red lines; interquartile range: box; 10*^th^*–90*^th^* percentiles: blue lines) as well as the individual values (circles or triangles) of the PID measures (**a**,**d**,**g**,**j**: joint information transfer; **b**,**e**,**h**,**k**: unique information transfer; **c**,**f**,**i**,**l**: synergistic and redundant transfer) computed at rest (R), during mental stress (M) and during serious game (G) considering the δ, θ, α, or β brain wave amplitude as the target process *j*, and the body and brain sub-networks as source processes *i* and *k*. Statistically significant differences between pairs of distributions are marked with * (R vs. M), with # (R vs. G), with *§* (R vs. R), with ∼ (M vs. M) and with ∘ (G vs. G).

**Figure 11:**
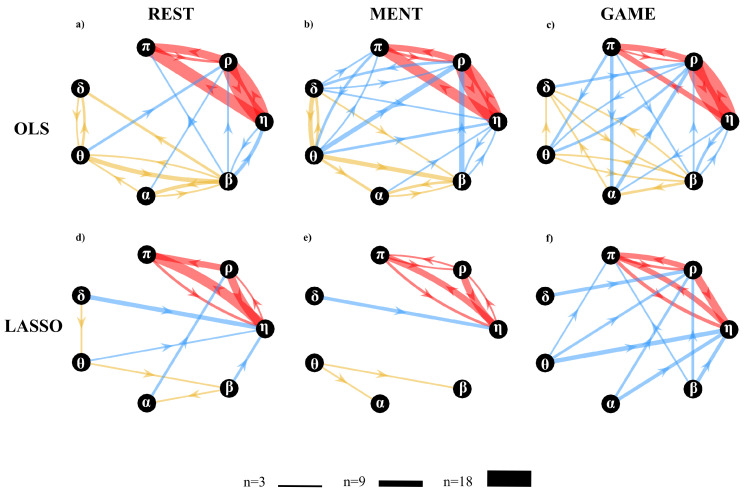
Topological structure for the networks of physiological interactions reconstructed during the three analyzes physiological states. Graphs depict significant directed interactions within the brain (yellow arrows) and body (red arrows) sub-networks as well as interactions between brain and body (blue arrows). Directed interactions were assessed counting the number of subjects for which the conditional transfer entropy ($T_{i\to j|s}$) was detected as statistically significant using OLS (**a**–**c**) or LASSO (**d**–**f**) to perform VAR model identification. The arrow thickness is proportional to the number of subjects (n) for which the link is detected as statistically significant.

**Figure 12:**
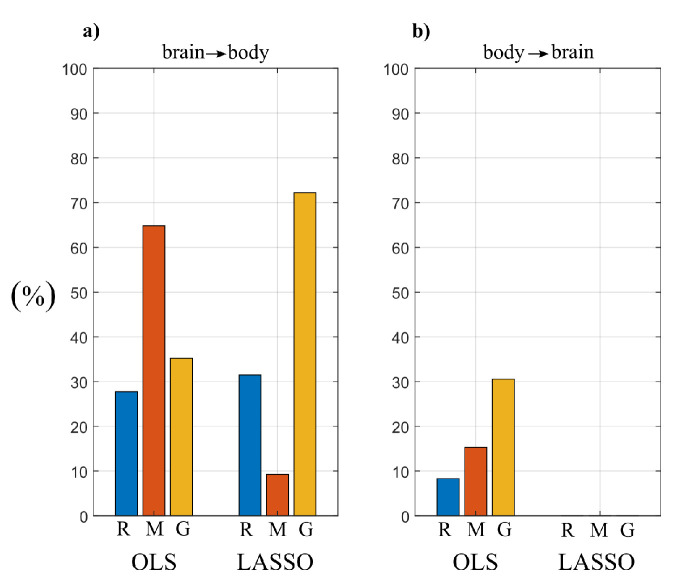
Bar plots reporting the in-strength index extracted from the cTE networks of Figure 11 by considering as link weights the percentage of subjects showing a brain-to-body connection (**a**) or a body-to-brain connection (**b**), computed at rest (R), during mental stress (M) and during serious game (G) for the two VAR identification methods. Please note that the in-strength computed along the direction from body to brain using LASSO is null in all conditions.

**Figure 13:**
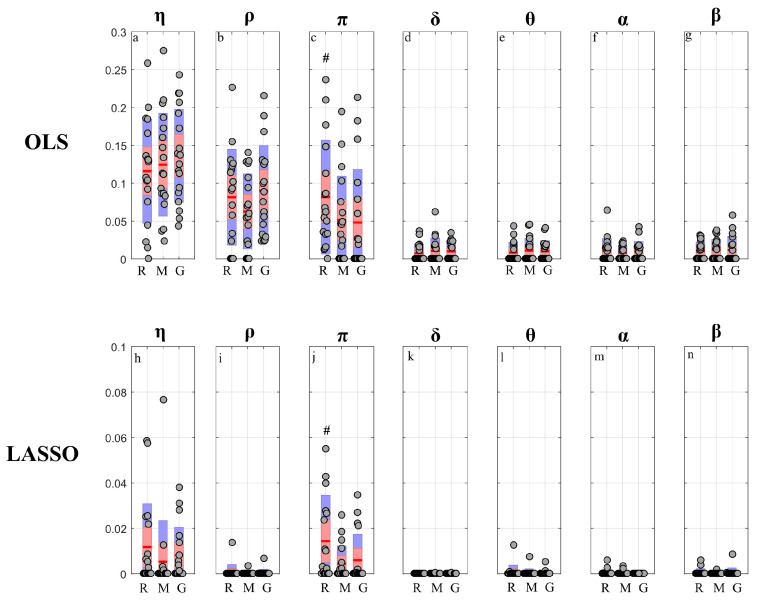
In-strength index computed for each node of the physiological network. Box plots report the distributions across subjects (median: red lines; interquartile range: box; 10*^th^*–90*^th^* percentiles: blue bars) as well as the individual values (circles) of the in-strength index (a-g) OLS, h-p LASSO) computed at rest (R), during mental stress (M) and during serious game (G) for each node (η,ρ,π,δ,θ,α,β). Statistically significant differences between pairs of distributions are marked with # (R vs. G).

The results of PID analysis, describing how information is transferred within the observed network of brain–body interactions, are reported respectively in Figure 7 (OLS results) and Figure 8 (LASSO results) for the targets belonging to the body sub-network (η,ρ,π), and in Figure 9 (OLS results) and Figure 10 (LASSO results) for the targets belonging to the brain sub-network (δ,θ,α,β). The results of cTE analysis, illustrating the topology of the detected physiological networks, are reported in Figure 11 (direct links), Figure 12 (brain–body interactions) and Figure 13 (in-strength). All analyses are performed identifying VAR models of dimension Mp, where M=7 and p∼4 (depending on the Bayesian Information Criterion) on time series of 300 points, which brought us to work with values K∼10 for the parameter relating the amount of data sample available to the model dimension.

#### 4.4.1. Partial Information Decomposition

Figure 7 and Figure 8 report, respectively for OLS and LASSO estimation, the distributions across subjects of the joint TE ($T_{ik\to j}$, left panels) directed to each target *j* belonging to the body sub-network from the two other body sources (index *i*) and from the four brain sources (index *k*), as well as of its decomposition into unique TEs ($U_{i\to j}$ and $U_{k\to j}$, middle panels) and redundant and synergistic TEs ($R_{ik\to j}$, $S_{ik\to j}$, right panels), evaluated at rest (R), during mental stress (M) and serious game (G).

Figure 7 shows that for each target in the body sub-network, the trends of the joint TE ($T_{ik\to j}$, Figure 7a,d,g) are mostly determined by the processes belonging to the same sub-network, as documented by the substantial values of the unique information transfer $U_{i\to j}$ and the negligible values of the unique transfer $U_{k\to j}$ (Figure 7b,e,h, with statistically significant difference between $U_{i\to j}$ and $U_{k\to j}$) and by the low values of the information transferred to η, ρ and π in a synergistic or redundant way from the brain and body sub-networks (Figure 7c,f,i). While for the targets η and ρ the PID measures did not vary significantly across conditions, the information transferred jointly from the brain and body sources towards the target π (Figure 7g) as well as the unique information transferred to π internally in the body sub-network (Figure 7h) decreased significantly moving from R to M and from R to G. This result documents a reduction of the causal interactions from RR interval and respiration towards the pulse arrival time during conditions of mental stress.

As reported in Figure 8, the trends of the joint TEs computed after LASSO identification when the processes η and π (a-g) are taken as target are comparable to those obtained with OLS identification and shown in Figure 7. In particular, also in this case a significant reduction of the joint TE directed to π is observed during the conditions M and G compared to R (Figure 8g), which is mostly due to a decrease of the unique information transferred to π from the body source ($U_{i\to j}$, Figure 8h). Moreover, also in this case the unique TE directed towards η and π from the brain sub-network ($U_{k\to j}$, Figure 8b,h) shows values very close to zero (b-h) and significantly lower than those of the unique TE $U_{i\to j}$. While the synergistic TE $S_{ik\to j}$ is almost zero for any target, the redundant TE $R_{ik\to j}$ is significantly higher than $S_{ik\to j}$ when the target is the vascular process π (Figure 8i). A result demonstrated specifically using the LASSO identification method is the absence of joint TE directed to the respiration process ρ (Figure 8d), documenting the absence of interactions directed toward respiration in all physiological conditions.

Figure 9 and Figure 10 report, respectively for OLS and LASSO estimation, the distributions across subjects of the joint TE ($T_{ik\to j}$, left panels) directed to each target *j* belonging to the brain sub-network from the three other brain sources (index *k*) and from the three body sources (index *i*), as well as of its decomposition into unique TEs ($U_{i\to j}$ and $U_{k\to j}$, middle panels) and redundant and synergistic TEs ($R_{ik\to j}$, $S_{ik\to j}$, right panels), evaluated at rest (R) and during mental stress (M) and serious game (G).

Considering the joint TE exchanged toward the brain rhythms, in contrast to what observed for the body sub-network (Figure 7a,e,g), the joint TE assessed through OLS identification shows a tendency to increase during M and especially during G compared to R (Figure 9 a,d,g,j); the increase is statistically significant for the δ (Figure 9a), and is supported by a significant increase of the redundant and synergistic TEs $R_{ik\to j}$ and $S_{ik\to j}$ which suggests an increased contribution of brain–body interactions to the rhythmic variations of the δ brain wave amplitude. An increase of the redundant brain–body interactions during stress states is observed also for the θ brain wave amplitude (Figure 9f). The analysis of the unique information transfer (Figure 9b,e,h,k) shows that the unique information provided by the brain sub-network ($U_{k\to j}$) is generally larger than that provided by the body sub-network ($U_{k\to j}$), with statistically significant differences during R and when the target of the unique transfer is given by the processes θ, α and β.

When PID directed towards the brain processes is computed using LASSO (Figure 10), a main result is that interactions are weak and do not vary significantly across physiological states. Notably, the joint TE and all PID terms relevant to the target δ are almost equal to zero in all conditions (Figure 10a,b,c). Similarly, also the values of the unique TE from the body sub-network to any brain process ($U_{i\to j}$, Figure 10b,e,h,k) and of both the redundant and synergistic TE ($R_{ik\to j}$, $S_{ik\to j}$, Figure 10c,f,i,l) are zero in almost all subjects and conditions, indicating that the LASSO approach does not detect interactions directed from body to brain in this dataset.

#### 4.4.2. Conditional Information Transfer

Figure 11 reports the network of physiological interactions reconstructed through the detection of the statistically significant values of the conditional transfer entropy ($T_{i\to j|s}$) computed for any pair of processes belonging to the brain and body sub-networks. The weighted arrows, depicting the most active connections among systems (arrows are present when at least 3 subjects show significant values of $T_{i\to j|s}$) show a similar structure when estimated in the three analyzed conditions using OLS (Figure 11a–c) and LASSO (Figure 11d–f). The main distinctive features are the existence of a densely connected sub-network of body interactions (red arrows), of a weakly connected sub-network of brain interactions (yellow arrows), and of changing patterns of brain–body interactions (blue arrows). In general, LASSO shows, for each condition analyzed, a greater sparsity in the estimated networks, preserving only the most active links detected by OLS.

Within body interactions are characterized mainly by cardiovascular links (interactions from η to π) and cardio-respiratory links (interactions between η and ρ), with a weaker coupling between ρ and π which exhibits a preferential direction from ρ to π; the use of LASSO elicits the unidirectional nature of cardio-respiratory interactions (from ρ to η). On the other hand, the topology of the brain sub-network is less stable in the three conditions and appears to lose consistency passing from REST to GAME; also in this case the use of LASSO leads to a greater sparsity, with nodes almost fully disconnected. As to brain–body interactions, they occur almost exclusively along the direction from brain to body; in this case the use of LASSO demonstrates that interactions from brain to body increase during the GAME condition.

To quantify the overall extent of the brain–body interactions from the above estimated cTE networks, was computed the percentage of subjects with statistically significant values of the cTE along the direction from brain to body and in the opposite direction from body to brain. This was obtained considering the brain sub-network and the body sub-network as single nodes, and computing the in-strength to one sub-network by considering only the connections coming from the other sub-network. The average values are shown in Figure 12.

The results reported in Figure 12 show that interactions are found more consistently along the direction from brain to body than along the opposite direction. In particular, LASSO does not show any link directed from body to brain in any of the three analyzed conditions. In the resting condition (R), the percentage of active links directed from brain to body is similar for the two VAR identification methods. Then, OLS identification results in a larger number of links moving from R to M, and a decrease during G. Conversely, LASSO shows a decrease of the percentage of significant links during M and a sharp increase during G.

Figure 13 reports the distribution of the values of the in-strength index evaluated for each node of the network in each experimental condition. For both OLS and LASSO, the median value of the in-strength index (Figure 13a–c,h–j) is higher for the network nodes of the body sub-network than for those belonging to the brain sub-network (Figure 13d–g,k–n). An exception to this difference is the in-strength of the links directed towards the node ρ, which is very close to zero when assessed using LASSO identification (Figure 13i). Moreover, the estimated in-strength values are, on average, lower when assessed through LASSO than through OLS. Considering the in-strength of individual nodes, a statistically significant reduction is observed moving from R to G for the weights of the connections directed towards π (Figure 13c,j), for both OLS and LASSO methods.

## 5. Discussion

### 5.1. Simulation Study I

The first simulation study was designed to compare the performance of the traditional OLS approach and the LASSO regression, implemented for the identification of VAR models in their state–space formulation [28], in estimating the information measures related to PID. The decomposition of the information transferred jointly from two sources to a target process allows investigation of how information is modified in a non-trivial way through redundant and synergistic interactions between the sources [64]. In particular, the model structure adopted in our simulation highlights the coexistence of synergistic and redundant contributions to the target $Y_{4}$ from the two sources $Y_{2}$ and $Y_{3}$ even if they are not directly coupled [30]. In situations such as this, the adoption of PID is fundamental to elicit how the two sources contribute to the target with both redundant and synergistic information transfer: the redundant contribution refers to the common information that both sources convey to the target; the synergistic contribution is considered an extra information transferred towards the target and is ascribed to the weakest source in the system [15].

The analysis in Figure 2 and Figure 3 shows an evident dependence of both the bias and the variance of all partial information decomposition measures on the factor *K*. This result is expected and reflects the well-known decrease of the prediction accuracy with the number of data samples available. In this context, our results document that the LASSO regression performs better in challenging conditions when the number of model parameters approaches the sample size (K≤5). In these conditions it has been pointed out how OLS is not suitable for the solution of a regression problem and that its solution could even not exist [40,65]. On the other hand, LASSO shows high robustness to the lack of data points, which results in limited values of bias and variance [66]. We note that despite this better performance of LASSO, in the condition K=1 all the PID measures that were different from zero ($T_{2\to 4}$,$T_{3\to 4}$,$S_{23\to 4}$,$R_{23\to 4}$) exhibit a consistent negative bias (Figure 2). This severe under estimation was previously highlighted in different scenarios, in which LASSO shrinkage produces biased estimation for the large coefficients and thus in some conditions could be sub-optimal in terms of estimation risk [67,68].

When the amount of data sample is not scarce compared to the number of model parameters (K>5) the performance of the two identification methods is comparable, with slight differences depending on the true value of the PID measures. In the case of non-zero PID measures (Figure 2) OLS showed better performance than LASSO in terms of bias and variance. This result is mainly due to the effect of the constraint based on the $l_{1}$ norm that performs a variable selection but with an increased bias and variance in the performed estimate [34,38].

On the other hand, in the scenario in which all the PID measures are equal to zero (Figure 3), LASSO performs better than OLS in all the conditions analyzed as regards both the bias and the variance of the estimates of information transfer. This can be explained with the continuous shrinkage and selection of the most relevant coefficients that set to zero most of the estimated AR coefficients [48].

### 5.2. Simulation Study II

The second simulation was designed to compare the performance of OLS and LASSO identification in estimating the cTE in a network of multiple interacting processes. The tested measure is highly relevant, as it is equivalent to the multivariate (conditional) Granger causality measure estimated within the most accurate framework available, i.e., that of vector state–space models [28]. Within this framework, we assessed both the statistical significance and the accuracy of the estimated values of the cTE, thus comparing OLS and LASSO regarding their accuracy in detecting the network structure and the coupling strength.

The accuracy in the estimation of the cTE values was investigated across different K ratio levels by means of BIAS and $BIAS_{N}$ used as performance parameters (Figure 5). As expected, both parameters show a tendency to increase as the *K* ratio decreases. This tendency is evident particularly for OLS estimation, as already documented testing different VAR parameter identification approaches (e.g., the Levinson recursion for the solution of Yule-Walker equations) in the context of signal processing [31]. The situation becomes worse when approaching the condition K=1, in which the matrix ${\left({\left[y^{p}\right]}^{T}y^{p}\right)}^{-1}$ approaches singularity. Consequently, in this case the solution to the DARE equation necessary to convert the SS model into the ISS form did not converge, thus impeding OLS-based estimation of the cTE. In such conditions it is necessary to move to the use of penalized regression techniques [34,38,40]. Here we document that the LASSO regression leads to trends of the cTE bias which are consistently very low for any value of *K* in the estimation of the null links (Figure 5a), and rise with *K* but without exhibiting abrupt increases even for K=1 in the estimation of the non-null links (Figure 5b). These good performances of LASSO identification confirm its higher tolerance to collinearity between regressors caused by the reduction of data samples available [69].

The reliability in the reconstruction of the network structure was investigated analyzing the performance of the two identification methods in terms of overall accuracy and rates of false negative and false positive detections. The ACC parameter appeared to be the best-suited indicator to synthesize the similarity between the estimated network and the ground-truth network [60]. Moreover, with the network structure simulated here, ACC is not affected by the class imbalance problem, a typical condition in sparse networks [70]. As expected, the ACC parameter decreased with the *K* ratio, with LASSO performing progressively better than OLS (Figure 6c). These results are in line with previous studies reporting the performance of different methods for the assessment of the statistical significance of causal interactions in different methodological contexts [33,34,56].

When the test was particularized to the rate of correct detection of null and non-null links, the performance under conditions of data paucity differ for the two identification methods, with LASSO showing better capability to correctly detect existing links (lower FNR) and OLS showing slightly better capability to correctly detect the absent links (lower FPR). In particular, by analyzing the trends of FNR (Figure 6a) LASSO showed better performance than OLS for K≤10, especially when the conditions for the estimation become very challenging (K≤5). This behavior is related to the shrinkage of the VAR parameters. In fact, the selected lambda tends to rise if the number of data samples decreases and this implies a greater sparsity of the estimated network with a high probability of producing false negatives [71]. In the same conditions, the value of FNR for OLS was around 60%. This poor performance is likely due to an inaccurate representation of the distribution of the cTE under the null hypothesis of uncoupling, estimated empirically using uncoupled surrogate time series, performed with very few data samples. On the contrary, while both methods display a low number of false positives for K>5, LASSO tends to produce an over-selection of the estimated links when K≤5. This result is in line with previous findings in the context of GC estimation, in which LASSO showed few extra links, observed for different combinations of degree of sparsity of the simulated network structure and *K* ratio [39,42].

### 5.3. Real Data Application

#### 5.3.1. Partial Information Decomposition Analysis

The main results of the partial decomposition of the information transfer within the network of brain and body interactions are that: (i) a significant information is transferred within the body sub-network, composed by the processes representative of the cardiac (η, heart period), vascular (π, pulse arrival time) and respiratory (ρ) dynamics, which is directed towards the η and π nodes as a result of respiration-related and cardiovascular effects; (ii) the information transferred to the nodes of the brain sub-network, representing the amplitude variations of the δ, θ, β, and α EEG waves, is lower and due almost exclusively to internal dynamics within this sub-network; (iii) a negligible amount of information is transferred between the two sub-networks as a result of their redundant or synergistic interaction. While these results are observed consistently using the two VAR identification methods (see Figure 7, Figure 8, Figure 9 and Figure 10, respectively), the use of the LASSO regression allows the elicitation of them more clearly. From a methodological point of view, this behavior is a result of the inclination towards sparseness of the LASSO method, which shrinks towards zero most of the VAR parameters that have a small effect on the target dynamics [38]. Such inclination puts also in evidence other behaviors, such as the substantial absence of information directed to the ρ node of the body network and to the δ node of the brain network. While in the first case the result is physiologically plausible since cardio-respiratory interactions are known to be almost unidirectional in nature (i.e., previous studies have found that respiration significantly affects the cardiovascular variables without being affected by them [2,57,72]), in the second case it could be related to an underestimation of the information transfer with the LASSO technique, since the δ waves seem to play a role in the organization of brain dynamics [1,7,73].

As the results reported above were observed consistently independently on the analyzed physiological state, they could be interpreted as a hallmark of how the networks of brain and body interactions organize their dynamic communication evaluated in terms of information transfer. Nevertheless, the conditions of mental stress evoked by the mental arithmetic task and the sustained attention task were able to induce, when compared with the resting condition set as baseline, some significant modifications in the amount of information transferred toward some specific nodes. In particular, a significant reduction of the joint brain–body TE computed when π was taken as the target process was observed during the two stress conditions compared to rest. This joint information transfer was due almost exclusively to contributions of unique transfer from the η and ρ nodes of the body sub-network (Figure 7h and Figure 8h), with a small amount of redundant brain–body information transfer (Figure 7h and Figure 8i) and negligible amounts of synergistic transfer or unique transfer from the brain sub-network; the unique transfer reflects cardiac and respiratory effects on the variability of the pulse arrival time, while the redundant transfer is related to common mechanisms whereby such variability is influenced by the brain rhythms one side and the cardio-respiratory rhythms on the other side. In this context, the results here obtained are in line with those obtained in [3] where a significant reduction of total information transferred towards π was found while playing a serious game with respect to a resting condition. Analyzing the same dataset in terms of mutual information, the authors of [44] found a significant reduction of the information shared between the pulse arrival time (π) and the cardio-respiratory system (η, ρ) during the conditions M and G compared with R. The significant decrease of the static mutual information computed in [44] and the dynamic measure of the joint and unique TE computed in the present study can be viewed as different aspects of the weakening of cardiovascular and cardio-respiratory interactions during mental stress. Physiologically, the underlying mechanisms could include an increased modulation of peripheral vascular resistance during stress which, as highlighted in [53,74], could dampen the modulation of the pulse arrival time due to heart rate variability and respiration.

When the target process belongs to the brain sub-network, the information transfer estimated through the LASSO regression was almost null when directed towards δ and very small when directed towards θ, α or β (Figure 10a–c). This result may reflect the lack or significant connectivity towards the brain sub-network, or the lower sensitivity of penalized regression methods to weak connectivity. In fact, using OLS a certain amount of information transfer to the nodes of the brain network was detected, with a significant increment of the joint transfer entropy from R to G when δ is the target process (Figure 9a), that is mostly due to the significant increment of redundant and synergistic TEs (Figure 9c). Furthermore, a significant increase of the redundant TE ($R_{ik\to j}$) was also observed during M and G with respect to R when θ is the target process (Figure 9f). The involvement of the brain waves during mental stress tasks was also investigated using information measures in [44], finding a larger involvement of δ and θ activity compared to rest that agrees with the results obtained here in terms of redundant TE computed after OLS identification.

#### 5.3.2. Conditional Information Transfer Analysis

The analysis of the statistically significant values of the conditional information transfer (cTE measure) led us to detect specific topology structures for the sub-networks that compose the overall physiological network of brain and body interactions (Figure 11). First, a quite consistent topology was found across different physiological states for the interactions between the cardiovascular and respiratory systems (Figure 11a–c and Figure 11d–f, red arrows), which is in line with a recent similar work performed in the context of information dynamics [3,18]. In particular, the strong link connection between η and ρ reflects a marked coupling between the heart rate variability and respiration, which is due to the well-known mechanisms such as respiratory sinus arrhythmia (RSA) [75] and cardio-respiratory synchronization [76]. This connection was detected as bidirectional using OLS, and as unidirectional from ρ to η using LASSO, confirming that the preferential direction of the cardio-respiratory interactions is that documenting the effect of respiration on the heart rate (RSA) [2,49,76]. Second, the information transferred from η to π reflects the well-known effect of the heart rate on stroke volume and arterial pressure which has a modulating effect on the arterial pulse wave velocity [77]. Moreover, the influence of respiration ρ on the pulse arrival time variability π reflects the breathing influences on the intra-thoracic pressure, blood pressure and blood flow velocity [77].

A further result relevant to the peripheral sub-network is the significant decrease of the in-strength relevant to the vascular node π observed for both OLS and LASSO moving from rest to the serious game condition independently (Figure 13c,j). This weaker topology is likely related to the significantly lower amount of information transferred towards π during the condition G compared to R (Figure 7g and Figure 8g). From a physiological point of view, this lower transfer mediated by weaker topology could suggest a reduction of the efferent nervous system activity from the cardiac and respiratory centers and directed towards the vascular system during conditions of mental attention.

Compared with the body sub-network, the links of the brain sub-network form a structure which seems less consistent across the different experimental conditions (Figure 11, yellow arrows). While OLS estimation shows an apparent decrease in the number of connections moving from R to M and especially to G, the LASSO regression yields an almost disconnected sub-network of brain-brain interactions. In contrast to that observed in this work, in [3] a more connected brain sub-network was found during the mental arithmetic task with respect to the resting condition. This difference can be partially methodological, as different model order selection criteria (Akaike vs. Bayesian) and methods to assess the statistical significance of cTE (F-test vs. surrogate data) were used in [3] and in the present work. These choices could indeed affect the estimation procedure and provide slightly different results especially in the presence of weak connections as in this case [56,78,79].

Finally, exploration of the network of dynamical interactions between the brain and the peripheral systems led us to investigate how the EEG dynamics, mostly determined by the central nervous system, interact with the cardiovascular and respiratory dynamics regulated by the autonomic nervous system (Figure 11, blue arrows, and Figure 12). Although quantitative statistical comparison cannot be performed for the results reported in Figure 11 and Figure 12 they document that brain–heart interactions are mostly oriented in the direction from brain to heart. This suggests that efferent autonomic commands directed to the peripheral systems follow in time the neural modulation of the brain wave amplitudes. Moreover, we find that the two mental stress conditions induce an enhancement of brain–body interactions, with a substantial increase of the number of significant links directed from the brain to the body sub-network and assessed using OLS during the mental arithmetic condition, or using LASSO during the serious game condition. The results based on OLS resemble those obtained recently on the same dataset [3], and recall previous findings highlighting significant correlations between the amplitude of brain oscillations (especially in the β band) and the heart rate and respiration dynamics [7,80]. The results based on LASSO highlight the emergence during sustained attention evoked by serious game playing of causal interactions from brain to the peripheral systems, mostly originating from the θ,α and β nodes and directed to the ρ and η nodes. These findings are supported by previous studies suggesting that the neural mechanisms responsible for the generation of α and θ brain oscillations are crucial for attention tasks and can be correlated with the cardiac autonomic activity and to its respiratory determinants [81,82,83].

## 6. Conclusions

The aim of this work was to test the usefulness of penalized regression techniques for the computation of different parametric measures of information transfer in networks of coupled stochastic processes. In particular, we considered the LASSO regression, a well-known technique that has been extensively used in different research fields, and implemented it for the first time within the most advanced framework for the linear parametric estimation of information dynamics, i.e., that based on the state–space computation of conditional Granger causality and partial information decomposition in vector stationary stochastic processes [15,28,30]. Our comparative validation with the traditional least squares identification of vector stochastic processes (OLS estimator) highlighted that LASSO allows highly accurate estimation of not only the amount of information transferred between coupled processes, but also the topological structure of the underlying network, especially in conditions of data paucity which make OLS estimation unreliable or even not applicable. On the other hand, in favorable conditions of data size related to the dimension of the model to be identified the results of classical and penalized regression were fully overlapped, confirming the appropriateness of embedding LASSO into the framework for the linear parametric analysis of information dynamics.

The application of the two identification methods to the study of the network of physiological interactions within and between brain and peripheral dynamics has demonstrated consistent patterns of information transfer and similar network structures. Here, the main findings regard the detection of significant information transfer within the body sub-network sustained by cardiovascular and respiratory dynamics, with reduced cardio-respiratory effects on the vascular dynamics in the presence of mental stress, and the existence of weak but significant brain–body interactions directed from the brain rhythms to the peripheral dynamics, with enhanced link strength in conditions of mental stress. It is worth noting that these results were obtained for K = 10, a condition in which the two identification procedures showed comparable performance in the simulation studies. This finding suggests that even in conditions that allow the use of OLS, LASSO is able to detect the strongest interactions among those determined by the combined activity of the central and autonomic nervous systems, providing as outcome estimated patterns of information dynamics which are more straightforward and easy to interpret than those obtained with OLS.

The directed links between different physiological systems observed in this study can reflect either well-defined physiological mechanisms, such as the respiratory and heart rate effects on the pulse arrival time [74,84], or statistical associations with likely common determinants of physiological origin, like the brain–heart interactions which are thought to be mediated by dynamic alterations of the sympatho-vagal balance [7,22,85]. In either case, approaches like ours that allow the probing of the dynamic interaction among different organ systems can be very useful to show how an imbalanced interaction may have a negative impact on health [85]. Previous studies have indeed demonstrated pathological changes in brain–body interactions with clinical significance, for instance related to sleep stages and insomnia [86], to sleep apneas [87] or to schizophrenia [72]. However, the analysis of brain–body interactions in different experimental conditions such as those analyzed in this paper, is somehow still unexplored and further studies need to be performed in order to strengthen the validity of the results obtained in the present and in previous studies.

Future developments will aim at testing the efficiency of different penalized regression techniques like those based on a linear combination of the $l_{1}$ and $l_{2}$ norms such as Elastic-net regression [88], or those based on a combination of OLS and LASSO such as adaptive LASSO, in order to overcome the problem related with the oracle property of LASSO [67]. Moreover, the comparison of penalized regression techniques with more specific approaches to dimensionality reduction in the computation of Granger causality and related measures [35,36] is envisaged to evaluate what approach is recommended for a reliable estimation of information dynamics in different conditions of network size and data length. Finally, future studies will also deal with the introduction of penalized regression techniques in the study of the information transfer within networks whose structure changes in time [89], or displaying dynamics which encompass multiple temporal scales [30].

## Figures and Tables

**Table 1:** F-values of the two-way repeated measures ANOVA. ** is associated with $p<10^{-5}$.

Factor	BIAS	${\mathrm{BIAS}}_{N}$	FNR	FPR	ACC
**K**	8582 **	1694 **	2204 **	197.2 **	2492 **
**TYPE**	1640 **	377 **	3538 **	223.4 **	1575 **
**K × TYPE**	8633 **	848 **	1055 **	114.5 **	339 **

## Supplementary Materials

Supplementary Material to this article is freely available for download from http://github.com/YuriAntonacci/PID-LASSO-toolbox and http://lucafaes.net/PIDlasso.html.
